# Allostatic load in thyroid cancer is higher than that of other cancers: A secondary analysis using NHANES

**DOI:** 10.1371/journal.pone.0341063

**Published:** 2026-01-22

**Authors:** Wen-rui Wang, Guan-dong Liu, Dong-xiao Ren, Xiao-yu Liu

**Affiliations:** 1 Department of Thyroid and Breast Surgery, Qilu Hospital of Shandong University Dezhou Hospital, Dezhou, Shandong, China; 2 Oncology Interventional Department, Qilu Hospital of Shandong University Dezhou Hospital, Dezhou, Shandong, China; 3 Department of Oncology, Qilu Hospital of Shandong University Dezhou Hospital, Dezhou, Shandong, China; Dynamical Business & Science Society - DBSS International SAS, COLOMBIA

## Abstract

**Background:**

To compare the levels of allostatic load score (ALS) between thyroid cancer patients and patients with other types of cancer and explore whether ALS mediates the association between thyroid cancer and alterations in physiological function.

**Methods:**

This cross-sectional study conducted a secondary analysis of 181 cancer patients using NHANES data from 2007 to 2020, including 91 individuals with thyroid cancer. Generalized linear regression, logistic regression, and sensitivity analysis were used to analyze the association between thyroid cancer and ALS in three different models. Receiver operating characteristic (ROC) curve analysis and feature importance analysis were utilized to assess the clinical predictive value of thyroid cancer. We also conducted a series of mediation analyses to examine the mediating role of ALS.

**Results:**

The ALS in thyroid cancer patients was higher than that in other cancer types (*P* < 0.05). Thyroid cancer was significantly associated with ALS even after adjusting for demographic variables (β = 0.770, 95%CI: 0.315–1.480; OR=2.255, 95%CI: 1.111–4.575). This association remained robust to missing data (all *P* < 0.05) and was not confounded by drinking, diabetes, or thyroid disease (all *P* > 0.05). Although thyroid cancer had limited predictive value on ALS, it exerted strong explanatory power. The mediation analysis conducted with imputed data and adjusted for confounding variables revealed that ALS fully mediated the effect of thyroid cancer on red cell distribution width (RDW) (IE: β = 0.103, *P* = 0.008; DE: β = 0.389, *P* = 0.056), with a mediation proportion of 20.93%.

**Conclusion:**

Our findings revealed that thyroid cancer condition were associated with elevated AL. AL mediated the relationship between thyroid cancer and RDW.

## 1. Introduction

Allostatic load (AL), a concept first proposed by McEwen and Stellar in 1993 [1], emphasizes the cumulative physiological burden of multiple systems resulting from prolonged adaptation to stressors. Allostatic load score (ALS), developed from key biomarkers, quantifies the effects of stress on the cardiovascular, metabolic, and immune systems [2,3]. Studies have highlighted that an increased ALS was associated with cardiovascular disease, cognitive function, and increased all-cause mortality [4–7], and ALS outperformed individual biomarkers in stress assessment and the associated biological burden [8]. High AL exerts cumulative wear and tear on multiple physiological systems (e.g., metabolic and cardiovascular systems) [4]. This impact manifests as dysregulation of physiological functions, such as impaired glucose metabolism and dysregulated inflammatory response [8]. Identifying individuals’ AL levels facilitates the implementation of targeted health intervention strategies.

Over the past several decades, the incidence of thyroid cancer has increased more rapidly than that of any other malignancy [9,10]. This epidemiological trend has resulted in a marked rise in the number of cancer survivors, given the disease’s 98.5% five-year survival rate [11]. Projections indicate that over one million individuals will be living with a thyroid cancer diagnosis within the next decade [12]. Based on its prognostic characteristics, thyroid cancer may impose less physiological strain in response to external stressors compared to other cancers. However, previous studies indicated that thyroid cancer survivors reported significantly lower quality of life than breast cancer survivors, while exhibiting similar outcomes to colon cancer survivors [13,14]. Moreover, thyroid cancer was associated with higher bankruptcy rates compared to other cancer types [15,16]. These findings starkly contradict the prevailing perception that thyroid cancer is a generally mild form of cancer. Consequently, compared to patients with other types of cancer, individuals with thyroid cancer may exhibit elevated ALS. As previously discussed, high ALS has been shown to exert detrimental effects on multiple physiological systems. Nevertheless, it remains to be determined whether the observed alterations in physiological system functions among thyroid cancer patients are mediated by ALS.

Therefore, we conducted a large-scale cross-sectional study to compare the levels of ALS between thyroid cancer patients and patients with other types of cancer and explore whether ALS mediates the association between thyroid cancer and alterations in physiological function. We hypothesized that compared with patients with other cancers, patients with thyroid cancer are associated with elevated ALS, and ALS may mediate the association between thyroid cancer and alterations in physiological function.

## 2. Methods

### 2.1. Data source and study participants

Secondary data were obtained from the National Health and Nutrition Examination Survey (NHANES), conducted by the U.S. Centers for Disease Control and Prevention (CDC), which is a nationally representative cross-sectional survey assessing health and nutritional status in the U.S. population. It combines interviews, physical examinations, and laboratory tests to collect data on demographics, diet, chronic diseases, biomarkers, and environmental exposures. All protocols used were approved by the Health Statistics Ethics Review Board of the U.S. CDC. All participants signed written informed consent forms, allowing anonymous data to be used for scientific research. This study followed the STandardisierte BerichtsROutine für Sekundärdaten Analysen (STROSA), which provide standardized guidelines for reporting secondary data analysis.

**Fig 1:**
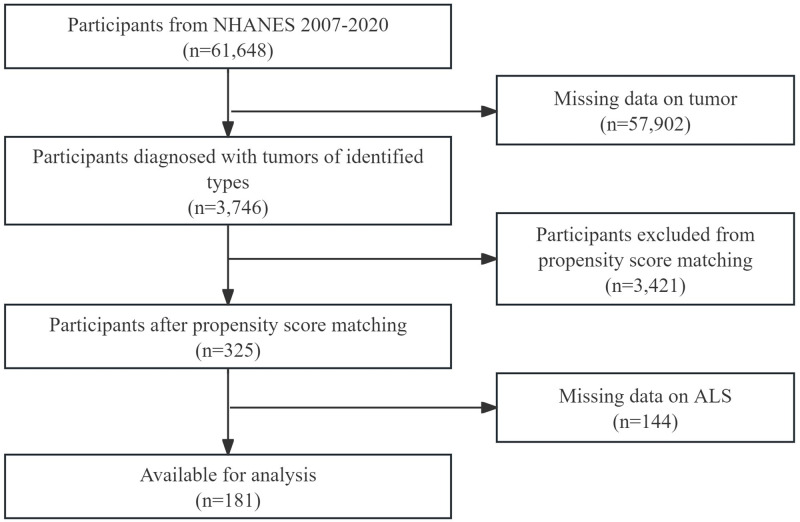
Flowchart of participants selection.

The most recent seven cycles of NHANES data available from 2007 to 2020 were included in this analysis. 61,648 participants were involved. Of these participants, we selected 3,746 individuals diagnosed with tumors of identified types. We employed propensity score matching (PSM) at a ratio of 1:3 to select patients with thyroid cancer and other cancers. Matching was performed using covariates including age, gender, race, education level, marital status, and poverty income ratio (PIR). After PSM, 325 individuals remained, including 88 samples with thyroid cancer and 237 samples with other types of cancer. Eventually, 181 participants with full ALS data and tumor data were enrolled in the study (Fig 1).

### 2.2. Definition of allostatic load score

AL was determined as an aggregate measure reflecting multisystem dysregulation, encompassing biomarkers related to cardiovascular, metabolic, and immune system [17]. Specifically, for the cardiovascular domain, systolic and diastolic blood pressure (SBP and DBP, mmHg), as well as total serum cholesterol (TC, mmol/L) and high-density lipoprotein cholesterol (HDL_C, mg/dL) levels, were incorporated. In terms of the metabolic system, glycohemoglobin (HbA1c, g/dL), albumin concentrations (g/dL), and body mass index (BMI, kg/m^2^) were included. For the immune system, C-reactive protein (CRP, mg/dL) levels were incorporated [18]. The eight biomarkers have been widely used in recent NHANES studies for the assessment of AL [3,18–20].

Based on prior research, ALS was calculated by converting each biomarker into a binary variable according to its statistical distribution within our dataset (using quartiles). If a biomarker fell within the highest quartile, it was assigned 1; otherwise, it received 0 (i.e., the lowest three quartiles). Exceptions were made for albumin and HDL_C, where lower values indicated higher risk. For these two variables, being in the lowest quartile resulted in 1 point being assigned. The possible ALS score ranged from 0 to 8, with a higher score indicating a stronger association of stress with physiologic dysregulation [3].

### 2.3. Assessment of cancer

Data on the cancer diagnosis and types were gathered from self-reported cancer assessments. Individuals who answered “Yes” to the question “Have you ever been told by a doctor or other health professional that you had cancer or a malignancy of any kind?” and “Thyroid” to the question “What kind of cancer was it?” were defined as thyroid cancer patients. Those who answered “Yes” to the first question but answered other types of cancer to the second were defined as patients with other types of cancer. In NHANES, the health questionnaire, physical examination, and biological sample collection were all completed on the same day at the Mobile Examination Center (MEC). Therefore, we can infer that the cancer diagnosis time of the study subjects must have occurred before the biomarker measurement. Some cycles of the NHANES dataset provide the age at the first diagnosis of thyroid cancer. We have listed the age information of the first cancer diagnosis for 9 thyroid cancer patients in this study from the 2007–2008 cycle in S1 Table for reference. The specific cancer types of the 181 patients enrolled in the analysis are presented in S1 Fig.

### 2.4. Covariates

This study incorporated covariates that may potentially influence the relationship between thyroid cancer and ALS, guided by previous research and clinical expertise: age, gender (male, female), race (Mexican American, Others), marital status (married, others), education level (under high school, high school or equivalent, above high school), PIR, smoking (yes, no), drinking (never, former, current), central obesity (yes, no), obesity (yes, no), hypertension (yes, no), diabetes (yes, no), thyroid disease (yes, no), liver disease (yes, no), hyperuricemia (yes, no), arthritis (yes, no), hypoalbuminemia (yes, no), gout (yes, no), menopause (yes, no), mean corpuscular hemoglobin concentration (MCHC, g/dL), and red cell distribution width (RDW, %).

Smoking status was classified as no (cotinine ≤15 ng/mL) or yes (cotinine >15 ng/mL). Drinking status was categorized as never (<12 lifetime drinks), former (≥12 lifetime drinks but no drinks in the last year), or current (drinks in the last 12 months) [21]. Obesity was defined as BMI ≥ 29.9 kg/m^2^. Hypertension was defined as a self-reported prior diagnosis, the use of antihypertensive medication, or a blood pressure reading of ≥140/90 mmHg. Diabetes was defined as a self-reported prior diagnosis, a fasting plasma glucose level of ≥7 mmol/L, a 2-h post-load plasma glucose level of ≥11.1 mmol/L, or the use of insulin or oral hypoglycemic medications. Hyperuricemia was defined as uric acid >7.0 mg/dL (male) or >6.0 mg/dL (female). Hypoalbuminemia was defined as albumin ≤35 d/L. Both MCHC and RDW are measured and calculated during the complete blood count (CBC) using the Beckman Coulter MAXM instrument. Serum cotinine is analyzed using isotope dilution-high-performance liquid chromatography coupled with atmospheric pressure chemical ionization tandem mass spectrometry (ID HPLC-APCI MS/MS). Fasting glucose and 2-hour post-load plasma glucose were determined via the hexokinase method. The serum uric acid concentration was quantified using the timed-endpoint method. Blood pressure levels were calculated as the mean of three consecutive readings obtained using a calibrated mercury sphygmomanometer, following a minimum five-minute rest period in a quiet room with a controlled ambient temperature. HbA1c was analyzed using a glycated hemoglobin analyzer. TC and HDL_C were assessed using a Roche Modular P chemistry analyzer. Albumin was measured via the bichromatic digital endpoint method. CRP was quantified through latex-enhanced nephelometry. BMI was calculated based on anthropometric measurements.

### 2.5. Statistical analysis

Continuous variables were normally distributed and expressed as mean and standard deviation (SD), while those with a skewed distribution were expressed as median and interquartile range (IQR). Categorical variables were expressed as number and percentage (%). Differences between groups were evaluated using the T-test or Mann-Whitney U-test for continuous variables and the Chi-square test or Fisher’s exact test for categorical variables. All statistical analyses were performed using SPSS version 26.0 and R version 4.2.1, and *P* values <0.05 (two-sided) were considered statistically significant.

Three different models were established to explore the association between thyroid cancer and ALS: the crude model (without adjusting for any covariates), Model 1 (adjusted for the PSM factors), and Model 2 (adjusted for the significant variables at baseline). Generalized linear regression was used to analyze the association between thyroid cancer and ALS in three different models. Based on its quartile distribution, ALS was dichotomized into a binary variable, with participants in the highest quartile classified as the high ALS group and those in the lower three quartiles categorized as the non-high ALS group. Logistic regression examines the relationship between one or several independent variables and a binary dependent variable [22]. Therefore, the relationship between thyroid cancer and binary variable ALS was investigated by three distinct logistic regression models. Diagnostic checks were performed to verify the adherence of all regression models to their respective statistical assumptions. In addition, we performed several sensitivity analyses to further validate the robustness of our results. Specifically, we examined whether the relationship between thyroid cancer and ALS was affected by missing data using two imputation methods (random forest and interpolation). Additionally, subgroup analyses were conducted to investigate whether other factors (drinking, diabetes, thyroid disease) might modify the association between thyroid cancer and ALS. The false discovery rate (FDR) correction for multiple comparison was performed.

Moreover, the clinical predictive value of thyroid cancer was assessed using receiver operating characteristic (ROC) curve analysis. Additionally, we employed logistic regression, XG Boost models, and SHapley Additive exPlanations (SHAP) values to evaluate the feature importance of thyroid cancer on ALS. SHAP enhances the interpretability of tree-based models by using a game-theoretic method to aggregate local feature contributions and explain global model behavior. This approach outperforms other global approximation methods and provides insights into both overall feature importance and specific prediction roles [23]. To further examine the mediating role of ALS, we performed a series of mediation analyses.

## 3. Results

### 3.1. Descriptive results

This study included 50 patients with thyroid cancer and 131 patients with other types of cancer, with a median age of 58 years and a female proportion of 80.11% (Table 1). Compared with survivors of other cancers, thyroid cancer survivors were more likely to have diabetes and thyroid disease (all *P* < 0.05). We also found a lower proportion of current drinkers in thyroid cancer participants (*P* < 0.05). Moreover, thyroid cancer patients exhibited higher levels of RDW, hs-CRP, HbA1c, and ALS, along with lower levels of MCHC (all *P* < 0.05). There was no significant difference in age, gender, race, education, marital status, PIR, BMI, smoking, central obesity, obesity, hypertension, liver disease, hyperuricemia, arthritis, hypoalbuminemia, gout, menopause, albumin, hemoglobin, CRP, HDL_C, and TC (all *P* > 0.05).

**Table 1:** Comparison of baseline characteristics according to the types of cancers.

Variable	Overall (n = 181)	Other cancers (n = 131)	Thyroid cancer (n = 50)	*P*-value
Age, years	58.000[46.000,68.000]	59.000[47.000,69.000]	54.000[46.000,67.000]	0.679
Gender, n (%)				0.660
Male	36(19.890)	25(19.084)	11(22.000)	
Female	145(80.110)	106(80.916)	39(78.000)	
Race, n (%)				0.847
Mexican American	31(17.127)	22(16.794)	9(18.000)	
Others	150(82.873)	109(83.206)	41(82.000)	
Education, n (%)				0.883
Under high school	23(12.707)	17(12.977)	6(12.000)	
High school or equivalent	40(22.099)	30(22.901)	10(20.000)	
Above high school	118(65.193)	84(64.122)	34(68.000)	
Marital status, n (%)				0.521
Married	109(60.221)	77(58.779)	32(64.000)	
Others	72(39.779)	54(41.221)	18(36.000)	
PIR	2.960[1.370,5.000]	2.980[1.360,5.000]	2.610[1.640,4.660]	0.681
BMI, kg/m^2^	28.000[24.700,33.600]	27.500[24.340,33.390]	28.210[25.830,35.000]	0.223
Smoking, n (%)				0.083
No	110(81.481)	73(77.660)	37(90.244)	
Yes	25(18.519)	21(22.340)	4(9.756)	
Drinking, n (%)				0.005
Never	15(8.621)	11(8.730)	4(8.333)	
Former	34(19.540)	17(13.492)	17(35.417)	
Current	125(71.839)	98(77.778)	27(56.250)	
Central obesity, n (%)				0.899
Yes	146(83.908)	106(84.127)	40(83.333)	
Obesity, n (%)				0.662
Yes	69(39.884)	49(38.889)	20(42.553)	
Hypertension, n (%)				0.199
Yes	91(50.276)	62(47.328)	29(58.000)	
Diabetes, n (%)				0.029
Yes	39(21.667)	23(17.557)	16(32.653)	
Thyroid disease, n (%)				<0.001
Yes	68(37.569)	21(16.031)	47(94.000)	
Liver disease, n (%)				0.104
Yes	10(5.525)	5(3.817)	5(10.000)	
Hyperuricemia, n (%)				0.323
Yes	45(24.862)	30(22.901)	15(30.000)	
Arthritis, n (%)				0.285
Yes	84(46.409)	64(48.855)	20(40.000)	
Hypoalbuminemia, n (%)				0.694
Yes	9(4.972)	6(4.580)	3(6.000)	
Gout, n (%)				0.865
Yes	10(8.197)	8(8.421)	2(7.407)	
Menopause, n (%)				0.887
Yes	48(36.090)	35(36.458)	13(35.135)	
MCHC, g/dL	33.715 ± 0.981	33.806 ± 0.931	33.476 ± 1.065	0.043
RDW, %	13.400[12.700,14.000]	13.300[12.500,13.900]	13.700[12.900,14.200]	0.030
Albumin, g/dL	4.200[3.900,4.400]	4.200[3.900,4.400]	4.100[3.900,4.200]	0.152
Hemoglobin, g/dL	13.600[12.700,14.400]	13.500[12.700,14.600]	13.600[12.500,14.300]	0.817
CRP, mg/dL	0.170[0.100,0.350]	0.170[0.100,0.350]	0.150[0.100,0.400]	0.826
hs-CRP, mg/L	2.500[1.260,5.500]	2.100[1.000,5.370]	3.900[1.810,8.670]	0.029
HbA1c, %	5.600[5.300,5.900]	5.500[5.300,5.800]	5.700[5.500,6.000]	0.014
HDL_C, mg/dL	1.370[1.090,1.730]	1.400[1.090,1.730]	1.320[1.010,1.730]	0.362
TC, mmol/L	4.970[4.220,5.720]	4.840[4.160,5.720]	5.120[4.320,5.640]	0.362
ALS	3.000[2.000,4.000]	2.000[1.000,4.000]	3.000[2.000,5.000]	0.011
ALS	2.762 (1.784)	2.550 ± 1.720	3.320 ± 1.845	0.009
ALS (categorical)				0.036
Low	134 (74.033)	103 (78.626)	31 (62.000)	
High	47 (25.967)	28 (21.374)	19 (38.000)	

Median [IQR] or mean ± SD for continuous variables and counts(percentage) for categorical variables. Abbreviations: IQR, interquartile range; SD, standard deviation; PIR, poverty income ratio; BMI, body mass index; MCHC, mean corpuscular hemoglobin concentration; RDW, red cell distribution width; CRP, C-reactive protein; hs-CRP, high-sensitivity C-reactive protein; HbA1c, hemoglobin A1c; HDL_C, high-density lipoprotein cholesterol; TC, total cholesterol; ALS, allostatic load score.

### 3.2. Association between thyroid cancer and ALS compared with other cancers

We employed generalized linear regression models to examine the association between thyroid cancer and ALS. We found that thyroid cancer was significantly associated with ALS even after adjusting for various covariates (Table 2). The unadjusted model (Crude model) yielded a β coefficient of 0.770 (95% confidence interval [CI]: 0.199–1.342), which remained significant in the adjusted models, with β coefficients of 0.898 (95% CI: 0.315–1.480) in Model 1 (adjusted for age, gender, race, education, marital status and PIR) and 0.881 (95% CI: 0.044–1.718) in Model 2 (adjusted for MCHC, RDW, drinking, diabetes and thyroid disease).

**Table 2:** Association between thyroid cancer and ALS compared with other cancers.

Model	ALS (continuous)		ALS (categorical)	
	β (95%CI)	*P*-value	OR (95%CI)	*P*-value
Crude model	0.770 (0.199, 1.342)	0.008	2.255 (1.111, 4.575)	0.024
Model 1	0.898 (0.315, 1.480)	0.003	2.600 (1.245, 5.431)	0.011
Model 2	0.881 (0.044, 1.718)	0.039	3.414 (0.948, 12.289)	0.060

Curde model: none of the variables were adjusted.

Model 1: adjusted for age, gender, race, marital status, education, and PIR.

Model 2: adjusted for MCHC, RDW, drinking, diabetes, and thyroid disease.

Abbreviations: ALS, allostatic load score; CI, confidence interval; OR, odds ratio; PIR, poverty income ratio; MCHC, mean corpuscular hemoglobin concentration; RDW, red cell distribution width.

After dichotomizing ALS using the highest quartile as the cutoff point, logistic regression models were established to investigate the association between thyroid cancer and ALS (Table 2). In the crude model, individuals with thyroid cancer exhibited a 2.255-fold increased risk of elevated ALS (odds ratio [OR]: 2.255, 95%confidence interval [CI]: 1.111–4.575). After adjusting for demographic confounders (Model 1), the association strengthened with an OR of 2.600 (95% CI: 1.245–5.431). Even the association did not exist in Model 2, with an OR of 3.414 (95%CI: 0.948–12.289), the *P* value was close to the threshold of 0.05, which suggested that the insignificant association may be caused by the relatively small sample size.

All assumptions for the linear regression models, including linearity, normality, and homoscedasticity, were met upon diagnostic testing. Similarly, the assumptions for the logistic regression models—including the absence of multicollinearity, linearity of independent variables and the log odds, and the lack of strongly influential outliers—were also satisfied. Detailed diagnostic plots and result of multicollinearity analysis are provided in the Supplementary Materials (S2-S3 Fig, S2 Table).

### 3.3. Sensitivity analysis and subgroup analysis

Random forest imputation captures complex patterns and handles non-linear relationships robustly, while interpolation offers simplicity and computational efficiency for structured data trends [24]. Thus, we employed these two imputation techniques to impute the missing data of 8 ALS-related variables in the remaining 325 individuals after PSM, to investigate whether the association between thyroid cancer and ALS was affected by missing data. The associations after data imputation remain consistent with the original results (Table 3). In the random forest approach, the crude model showed a significant positive association between thyroid cancer and ALS (β = 0.409, 95%CI: 0.014–0.804), which strengthened in Model 1 (β = 0.507, 95%CI: 0.106–0.909) and further increased in Model 2 (β = 0.690, 95%CI: 0.076–1.304). A similar pattern emerged using interpolation imputation, with borderline significance in the crude model (β = 0.375, *P* = 0.056) transitioning to statistically significant estimates in Model 1 (β = 0.486, *P* = 0.015) and Model 2 (β = 0.627, *P* = 0.038). Imputation of missing data increases the likelihood of non-significant findings for the crude model (*P* = 0.056).

**Table 3:** Association between thyroid cancer and ALS after imputation.

Imputation method	Model	ALS (continuous)	
		β (95%CI)	*P*-value
Random Forest	Crude model	0.409 (0.014, 0.804)	0.042
	Model 1	0.507 (0.106, 0.909)	0.013
	Model 2	0.690 (0.076, 1.304)	0.028
Interpolation	Crude model	0.375 (−0.010, 0.760)	0.056
	Model 1	0.486 (0.093, 0.880)	0.015
	Model 2	0.627 (0.034, 1.220)	0.038

Curde model: none of the variables were adjusted.

Model 1: adjusted for age, gender, race, marital status, education, and PIR.

Model 2: adjusted for MCHC, RDW, drinking, diabetes, and thyroid disease.

Abbreviations: ALS, allostatic load score; CI, confidence interval; OR, odds ratio; PIR, poverty income ratio; MCHC, mean corpuscular hemoglobin concentration; RDW, red cell distribution width.

Moreover, subgroup analysis was utilized to examine whether the association between thyroid cancer and ALS was modified by drinking, diabetes, and thyroid diseases (as these 3 indicators showed difference at baseline). As shown in Table 4, we found that the association between thyroid cancer and ALS was significant among current drinkers (OR=3.06, 95%CI: 1.21–7.69) and subgroups with comorbid diabetes (OR=6.00, 95%CI: 1.46–24.73) and thyroid diseases (OR=6.45, 95%CI: 1.34–30.95). Notably, this significant association remained statistically significant after FDR correction. There was no interaction effect among these three variables and thyroid cancer on ALS (all *P* for interaction >0.05), highlighting the stability of their association.

**Table 4:** Subgroup analyses on the association between thyroid cancer and ALS.

Subgroups	No.	OR (95%CI)	*P-*value	FDR	*P* for interaction
Drinking					0.403
Never	15	1.50 (0.10,23.07)	0.771	1.000	
Former	34	1.00 (0.26, 3.92)	1.000	1.000	
Current	125	3.06 (1.21, 7.69)	0.018	0.047	
Diabetes					0.086
Yes	39	6.00 (1.46, 24.73)	0.013	0.047	
No	141	1.39 (0.57, 3.39)	0.474	0.830	
Thyroid disease					0.985
Yes	68	6.45 (1.34, 30.95)	0.020	0.047	
No	113	0.00 (0.00, inf)	0.991	1.000	

Abbreviations: ALS, allostatic load score; OR, odds ratio; CI, confidence interval.

### 3.4. ROC curve analysis and Importance analysis

**Fig 2:**
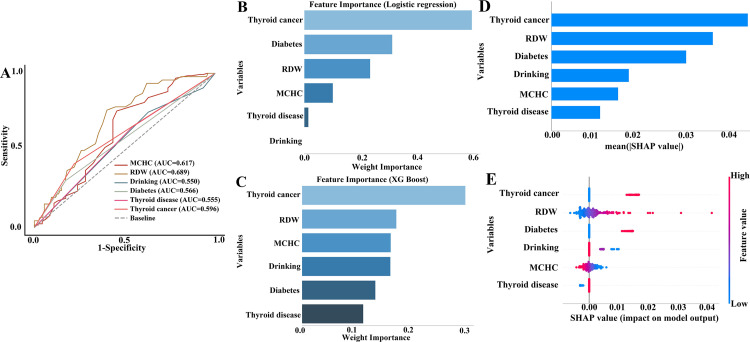
Receiver operating characteristic (ROC) curve analysis and importance analysis. **(A)** ROC plot; **(B)** Feature importance analysis by logistic regression model; **(C)** Feature importance analysis by XG Boost model; **(D)** SHAP feature importance plot; **(E)** SHAP summary plot. Abbreviations: MCHC, mean corpuscular hemoglobin concentration; RDW, red cell distribution width.

To explore the performance of thyroid cancer in predicting ALS levels, variables with significant differences in the baseline table were included. ROC curve analysis revealed that RDW demonstrated the highest area under the curve (AUC) 0.689, followed by MCHC (AUC: 0.617), and thyroid cancer (AUC: 0.596). Diabetes (AUC: 0.566), thyroid disorder (AUC: 0.555), and drinking (AUC: 0.550) showed relatively lower predictive values (Fig 2A).

Feature importance analysis was conducted using different models. In the logistic regression model, thyroid cancer ranked first, followed by diabetes, RDW, MCHC, thyroid diseases, and drinking (Fig 2B). In the XG Boost model, thyroid cancer also ranked first, followed by RDW, MCHC, drinking, diabetes, and thyroid diseases (Fig 2C). Feature importance analysis based on SHAP values revealed that thyroid cancer ranked first, followed by RDW, diabetes, drinking, MCHC, and thyroid disease (Fig 2D, 2E). These results suggested that thyroid cancer exerted strong explanatory power but has limited discriminatory capacity on ALS.

### 3.5. Mediation effects of ALS on the relationship of thyroid cancer with MCHC, RDW, and diabetes

**Fig 3:**
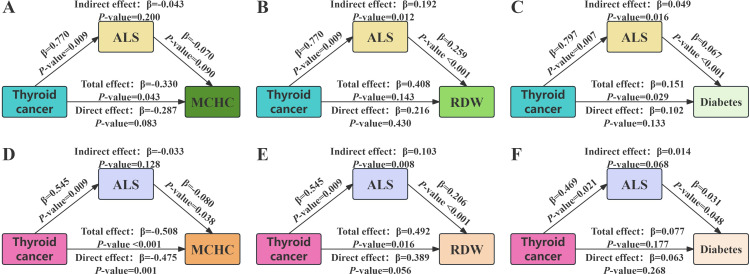
ALS mediation pathways between thyroid cancer and bodily functions. **(A)** MCHC; **(B)** RDW; **(C)** Diabetes; **(D-F)** Imputed data analyses (adjusted for age, gender, race, education, marital status, and PIR): **(D)** MCHC; **(E)** RDW; **(F)** Diabetes. Abbreviations: ALS, allostatic load score; MCHC, mean corpuscular hemoglobin concentration; RDW, red cell distribution width; PIR, poverty income ratio.

The main analysis revealed that patients with thyroid cancer exhibited higher ALS compared to individuals with other types of cancer. Given that elevated ALS has been shown to impair the functioning of multiple physiological systems, we further investigated whether the relationship between thyroid cancer and alterations in physiological functions is mediated by ALS. We selected variables that demonstrated significant intergroup differences in the baseline analysis and were pathophysiologically associated with various physiological systems—including MCHC, RDW, and diabetes—as biomarkers to assess changes in physiological function. Fig 3A and 3C showed thyroid cancer was associated with MCHC and diabetes (*P* for total effect [TE] <0.05), and ALS acted as a full mediator (indirect effect [IE]: β = 0.049, *P* = 0.016; direct effect [DE]: β = 0.102, *P* = 0.133) in thyroid cancer-diabetes pathway, accounting for 32.45% of the mediation effect. We observed that ALS exhibited a significant mediating effect in certain pathways, despite the total effect being non-significant (Fig 3B).

To enhance the robustness of our findings, we re-conducted the mediation analysis by increasing the sample size, utilizing imputed data, and adjusting for potential confounding factors (including age, gender, race, education level, marital status, and PIR). As illustrated in the participant selection flowchart (Fig 1), 144 individuals were excluded after PSM due to missing data on 8 ALS-related variables required for calculation. To maximize the sample size, we performed multiple imputation for the missing ALS-related variables, resulting in an imputed dataset comprising 325 participants. The results showed that ALS did not act as a mediator in the thyroid cancer-MCHC pathway (Fig 3D, *P* for IE > 0.05), but served as a complete mediator in the thyroid cancer-RDW pathway (Fig 3E; IE: β = 0.103, *P* = 0.008; DE: β = 0.389, *P* = 0.056). However, the analysis of imputed data failed to detect any significant influence of thyroid cancer on diabetes (Fig 3F, *P* for TE > 0.05).

## 4. Discussion

This study found that thyroid cancer was significantly associated with ALS even after adjusting for covariates. This association remained robust to missing data and was not confounded by drinking, diabetes, or thyroid disease. Although thyroid cancer did not demonstrate the best predictive value in the ROC curve analysis, it exerted strong explanatory power. Finally, the mediation analysis confirmed that ALS served as a complete mediator in the thyroid cancer-RDW pathway.

Compared with other types of cancer, thyroid cancer exhibited a robust association with elevated AL. Cancer and its treatment introduce numerous physiologic, psychological, social, and economic stressors to the individual with the diagnosis. Although thyroid cancer generally has a favorable prognosis, a systematic review indicated that the quality of life of thyroid cancer survivors was not higher than that of patients with other cancers; instead, it is lower than that of some malignant tumors, such as breast cancer [14]. Many patients feel deprived of support from their families and doctors because thyroid cancer is regarded by medical personnel as a “good kind of cancer”. This perception may underestimate the severity of the diagnosis and thereby increase AL [13]. Furthermore, patients receiving radioactive iodine (RAI) treatment or insufficient doses of levothyroxine may develop hypothyroidism. This condition can lead to a broad spectrum of symptoms, such as fatigue, altered appetite, disrupted sleep patterns, anxiety, and reduced therapeutic efficacy, all of which can significantly elevate AL [25]. The fear of cancer recurrence, concern over the risk of a second primary cancer, and anxiety regarding distant metastasis can impose significant psychological stress on thyroid cancer survivors, despite having received definitive treatment and potentially being in a disease-free state [26].

This study demonstrated that thyroid cancer could elevate RDW levels by increasing AL. Thyroid cancer elevates RDW by increasing AL through multiple pathways (inflammation, metabolic dysregulation, oxidative stress, etc.), which disrupt erythropoiesis and red cell metabolism. First, thyroid cancer may trigger chronic inflammation, releasing pro-inflammatory cytokines that suppress bone marrow erythropoiesis, leading to impaired red blood cell production and increased size heterogeneity, thereby elevating RDW [27–29]. Second, iron-deficiency anemia is a common cause of elevated RDW, which can be exacerbated by impaired nutrient absorption or drug side effects in thyroid cancer [30]. Third, thyroid cancer and its treatments may increase oxidative stress, damaging red blood cell membranes and shortening their lifespan, resulting in greater size variability and elevated RDW [31,32]. Four, abnormal thyroid hormone levels disrupt erythropoiesis by altering bone marrow microenvironments or erythropoietin signaling [33,34]. Lastly, thyroid cancer patients often have autoimmune comorbidities where autoantibodies may attack erythrocyte precursors or disrupt iron metabolism, contributing to elevated RDW [35]. These mechanisms are closely linked to the pathophysiology of the disease and treatment-related side effects.

The findings of this exploratory study highlight the potential clinical relevance of AL in thyroid cancer survivorship. These findings suggest that assessing AL might identify patients at higher risk of physiological dysregulation and could represent a promising target for future interventions aimed at improving long-term outcomes. However, before any clinical application can be considered, further research is essential to validate these findings and to establish standardized, feasible, and clinically relevant biomarker panels for measuring AL in routine practice.

Future longitudinal studies may help to understand whether AL is the cumulative result of the cancer burden. Such studies will also be helpful in examining the AL trajectory to enhance the understanding of susceptibility to cancer progression and mortality [36]. Comprehensive research is essential to elucidate how the interplay among genetics, psychology, physiology, behavior, and environment influences disease risk and treatment response [37]. Furthermore, future studies should aim to identify the optimal combination of biomarkers across various bodily systems for accurately quantifying AL, thereby enhancing comparability between studies [38].

This study has several limitations. First, the cross-sectional design precludes establishing temporal relationships between exposures and outcomes, thereby limiting causal inferences. Second, biomarker data from NHANES were available only at a single time point, which prevented the investigation of longitudinal changes or trajectories in ALS. Additionally, due to the relatively limited sample size in this study, certain subgroups within the subgroup analysis had particularly small sample sizes, leading to insufficient statistical power. As a result, the conclusions derived from these subgroup analyses should be interpreted cautiously. Future research should aim to validate our findings in larger and more representative cohorts. Moreover, cancer diagnosis and type were self-reported by participants, introducing potential recall and reporting biases. The study population comprised American adults, which introduces geographical and ethnic limitations, thereby restricting the generalizability of the findings to populations in other regions. Finally, residual confounding due to unmeasured variables cannot be entirely ruled out.

## 5. Conclusion

This study demonstrated a robust association between thyroid cancer and ALS. Despite thyroid cancer has limited discriminatory capacity on ALS level, it exerted strong explanatory power. Mediation analyses revealed ALS as a key mediator between thyroid cancer and RDW. Collectively, our findings position ALS as a significant risk marker and a potential biological pathway through which thyroid cancer may influence physiological dysregulation, offering a conceptual framework for understanding the long-term health burdens in this patient population.

## Supporting information

S1 Fig The distribution of cancer types. (TIF)

S2 Fig Diagnostic plots for the linear regression model. (TIFF)

S3 Fig Diagnostic plots for the logistic regression model. (TIFF)

S1 Table Time information for 9 patients with thyroid cancer in this study from the 2007–2008 cycle. (DOCX)

S2 Table Multicollinearity analysis between variables. (DOCX)

S1 File Raw data. (XLSX)

## References

[1] McEwen BS, Stellar E. Stress and the individual. Mechanisms leading to disease. Arch Intern Med. 1993;153(18):2093–101. 8379800

[2] Lai KY, Kumari S, Gallacher J, Webster CJ, Sarkar C. Association between Residential Greenness and Allostatic Load: A Cohort Study. Environ Sci Technol. 2024;58(11):4884–93. doi: 10.1021/acs.est.3c04792 38437596

[3] Dai Z, Zhou X. Associations between allostatic load and hepatic steatosis and liver fibrosis: evidence from NHANES 2017-2020. BMC Public Health. 2024;24(1):1602. doi: 10.1186/s12889-024-19111-7 38879469 PMC11179389

[4] Guidi J, Lucente M, Sonino N, Fava GA. Allostatic Load and Its Impact on Health: A Systematic Review. Psychother Psychosom. 2021;90(1):11–27. doi: 10.1159/000510696 32799204

[5] López-Cepero A, McClain AC, Rosal MC, Tucker KL, Mattei J. Examination of the Allostatic Load Construct and Its Longitudinal Association With Health Outcomes in the Boston Puerto Rican Health Study. Psychosom Med. 2022;84(1):104–15. doi: 10.1097/PSY.0000000000001013 34581702 PMC8678200

[6] Gillespie SL, Anderson CM, Zhao S, Tan Y, Kline D, Brock G, et al. Allostatic load in the association of depressive symptoms with incident coronary heart disease: The Jackson Heart Study. Psychoneuroendocrinology. 2019;109:104369. doi: 10.1016/j.psyneuen.2019.06.020 31307010 PMC7232849

[7] Zhang T, Yan LL, Chen H-S, Jin H-Y, Wu C. Association between allostatic load and mortality among Chinese older adults: the Chinese Longitudinal Health and Longevity Study. BMJ Open. 2021;11(8):e045369. doi: 10.1136/bmjopen-2020-045369 34344673 PMC8336121

[8] Juster R-P, McEwen BS, Lupien SJ. Allostatic load biomarkers of chronic stress and impact on health and cognition. Neurosci Biobehav Rev. 2010;35(1):2–16. doi: 10.1016/j.neubiorev.2009.10.002 19822172

[9] Kilfoy BA, Zheng T, Holford TR, Han X, Ward MH, Sjodin A. International patterns and trends in thyroid cancer incidence, 1973-2002. Cancer Causes Control. 2009;20(5):525–31. 19016336 10.1007/s10552-008-9260-4 PMC2788231

[10] Kilfoy BA, Devesa SS, Ward MH, Zhang Y, Rosenberg PS, Holford TR, et al. Gender is an age-specific effect modifier for papillary cancers of the thyroid gland. Cancer Epidemiol Biomarkers Prev. 2009;18(4):1092–100. doi: 10.1158/1055-9965.EPI-08-0976 19293311 PMC2667567

[11] Boucai L, Zafereo M, Cabanillas ME. Thyroid Cancer: A Review. JAMA. 2024;331(5):425–35. 38319329 10.1001/jama.2023.26348

[12] Davies L, Welch HG. Thyroid cancer survival in the United States: observational data from 1973 to 2005. Arch Otolaryngol Head Neck Surg. 2010;136(5):440–4. doi: 10.1001/archoto.2010.55 20479371

[13] Aschebrook-Kilfoy B, James B, Nagar S, Kaplan S, Seng V, Ahsan H, et al. Risk Factors for Decreased Quality of Life in Thyroid Cancer Survivors: Initial Findings from the North American Thyroid Cancer Survivorship Study. Thyroid. 2015;25(12):1313–21. doi: 10.1089/thy.2015.0098 26431811 PMC4684649

[14] Applewhite MK, James BC, Kaplan SP, Angelos P, Kaplan EL, Grogan RH, et al. Quality of Life in Thyroid Cancer is Similar to That of Other Cancers with Worse Survival. World J Surg. 2016;40(3):551–61. doi: 10.1007/s00268-015-3300-5 26546191

[15] Lubitz CC, Kong CY, McMahon PM, Daniels GH, Chen Y, Economopoulos KP, et al. Annual financial impact of well-differentiated thyroid cancer care in the United States. Cancer. 2014;120(9):1345–52. doi: 10.1002/cncr.28562 24481684 PMC3999178

[16] Ramsey S, Blough D, Kirchhoff A, Kreizenbeck K, Fedorenko C, Snell K, et al. Washington State cancer patients found to be at greater risk for bankruptcy than people without a cancer diagnosis. Health Aff (Millwood). 2013;32(6):1143–52. doi: 10.1377/hlthaff.2012.1263 23676531 PMC4240626

[17] Frei R, Haile SR, Mutsch M, Rohrmann S. Relationship of Serum Vitamin D Concentrations and Allostatic Load as a Measure of Cumulative Biological Risk among the US Population: A Cross-Sectional Study. PLoS One. 2015;10(10):e0139217. doi: 10.1371/journal.pone.0139217 26451600 PMC4599851

[18] Du EY, Jiang K, Carlson MC, Reed NS, Deal JA. Hearing Impairment and Allostatic Load in Older Adults. JAMA Otolaryngol Head Neck Surg. 2023;149(7):597–606. doi: 10.1001/jamaoto.2023.0948 37200015 PMC10196929

[19] Johnson NB, Jones EM, Ovbiagele B, Markovic D, Towfighi A. Effects of Allostatic Load on Long-Term Survival After Stroke. Stroke. 2025;56(1):87–94. doi: 10.1161/STROKEAHA.124.046622 39676664

[20] Li C, Howard SP, Rogers CR, Andrzejak S, Gilbert KL, Watts KJ, et al. Allostatic Load, Educational Attainment, and Risk of Cancer Mortality Among US Men. JAMA Netw Open. 2024;7(12):e2449855. doi: 10.1001/jamanetworkopen.2024.49855 39656456 PMC11632542

[21] Wang M, Lu X, Zheng X, Xu C, Liu J. The relationship between sleep duration and thyroid function in the adult US population: NHANES 2007-2012. PLoS One. 2023;18(9):e0291799. doi: 10.1371/journal.pone.0291799 37733750 PMC10513250

[22] Elkahwagy DMAS, Kiriacos CJ, Mansour M. Logistic regression and other statistical tools in diagnostic biomarker studies. Clin Transl Oncol. 2024;26(9):2172–80. doi: 10.1007/s12094-024-03413-8 38530558 PMC11333519

[23] Qi X, Wang S, Fang C, Jia J, Lin L, Yuan T. Machine learning and SHAP value interpretation for predicting comorbidity of cardiovascular disease and cancer with dietary antioxidants. Redox Biol. 2025;79:103470. doi: 10.1016/j.redox.2024.103470 39700695 PMC11729017

[24] Ou H, Yao Y, He Y. Missing data imputation method combining random forest and generative adversarial imputation network. Sensors (Basel). 2024;24(4). 10.3390/s24041112 PMC10893362 38400270

[25] Dow KH, Ferrell BR, Anello C. Quality-of-life changes in patients with thyroid cancer after withdrawal of thyroid hormone therapy. Thyroid. 1997;7(4):613–9. doi: 10.1089/thy.1997.7.613 9292951

[26] Lee JI, Kim SH, Tan AH, Kim HK, Jang HW, Hur KY, et al. Decreased health-related quality of life in disease-free survivors of differentiated thyroid cancer in Korea. Health Qual Life Outcomes. 2010;8:101. doi: 10.1186/1477-7525-8-101 20840792 PMC2949818

[27] Guarino V, Castellone MD, Avilla E, Melillo RM. Thyroid cancer and inflammation. Mol Cell Endocrinol. 2010;321(1):94–102. doi: 10.1016/j.mce.2009.10.003 19835928

[28] Tafani M, De Santis E, Coppola L, Perrone GA, Carnevale I, Russo A, et al. Bridging hypoxia, inflammation and estrogen receptors in thyroid cancer progression. Biomed Pharmacother. 2014;68(1):1–5. doi: 10.1016/j.biopha.2013.10.013 24286852

[29] Marzouk H, Mostafa N, Khalifa I, Badawi N, Mohamed Fathy Sabry NI. Red Cell Distribution Width (RDW) as a Marker of Subclinical Inflammation in Children with Familial Mediterranean Fever. Curr Rheumatol Rev. 2020;16(4):298–303. 32164513 10.2174/1573397116666200312142709

[30] Aulakh R, Sohi I, Singh T, Kakkar N. Red cell distribution width (RDW) in the diagnosis of iron deficiency with microcytic hypochromic anemia. Indian J Pediatr. 2009;76(3):265–8. doi: 10.1007/s12098-009-0014-4 19205647

[31] Joosse H-J, van Oirschot BA, Kooijmans SAA, Hoefer IE, van Wijk RAH, Huisman A, et al. In-vitro and in-silico evidence for oxidative stress as drivers for RDW. Sci Rep. 2023;13(1):9223. doi: 10.1038/s41598-023-36514-5 37286717 PMC10247684

[32] Muzza M, Pogliaghi G, Colombo C, Carbone E, Cirello V, Palazzo S, et al. Oxidative Stress Correlates with More Aggressive Features in Thyroid Cancer. Cancers (Basel). 2022;14(23):5857. doi: 10.3390/cancers14235857 36497339 PMC9737385

[33] Baliram R, Latif R, Zaidi M, Davies TF. Expanding the Role of Thyroid-Stimulating Hormone in Skeletal Physiology. Front Endocrinol (Lausanne). 2017;8:252. doi: 10.3389/fendo.2017.00252 29042858 PMC5632520

[34] Bebeshko VG, Bruslova KM, Boyarska OY, Lyashenko LO, Tsvyetkova NM, Gonchar LO, et al. Endocrine regulation of erythroid lineage of hematopoiesis in children living under a low-dose radiation exposure after the chornobyl npp accident. Probl Radiac Med Radiobiol. 2020;25:374–89. doi: 10.33145/2304-8336-2020-25-374-389 33361848

[35] Antonelli A, Ferrari SM, Corrado A, Di Domenicantonio A, Fallahi P. Autoimmune thyroid disorders. Autoimmun Rev. 2015;14(2):174–80. doi: 10.1016/j.autrev.2014.10.016 25461470

[36] Mathew A, Doorenbos AZ, Li H, Jang MK, Park CG, Bronas UG. Allostatic Load in Cancer: A Systematic Review and Mini Meta-Analysis. Biol Res Nurs. 2021;23(3):341–61. doi: 10.1177/1099800420969898 33138637 PMC8755951

[37] Bu S, Li Y. Physical activity is associated with allostatic load: Evidence from the National Health and Nutrition Examination Survey. Psychoneuroendocrinology. 2023;154:106294. doi: 10.1016/j.psyneuen.2023.106294 37216739

[38] Rosemberg M-AS, Granner J, Li Y, Seng JS. A scoping review of interventions targeting allostatic load. Stress. 2020;23(5):519–28. doi: 10.1080/10253890.2020.1784136 32602798 PMC7841966

