# Supplementary figures and images for: Allostatic load in thyroid cancer is higher than that of other cancers: A secondary analysis using NHANES
Source: PLoS One. 2026 Jan 22;21(1):e0341063. doi: 10.1371/journal.pone.0341063 (PMC12826484; doi:10.1371/journal.pone.0341063)

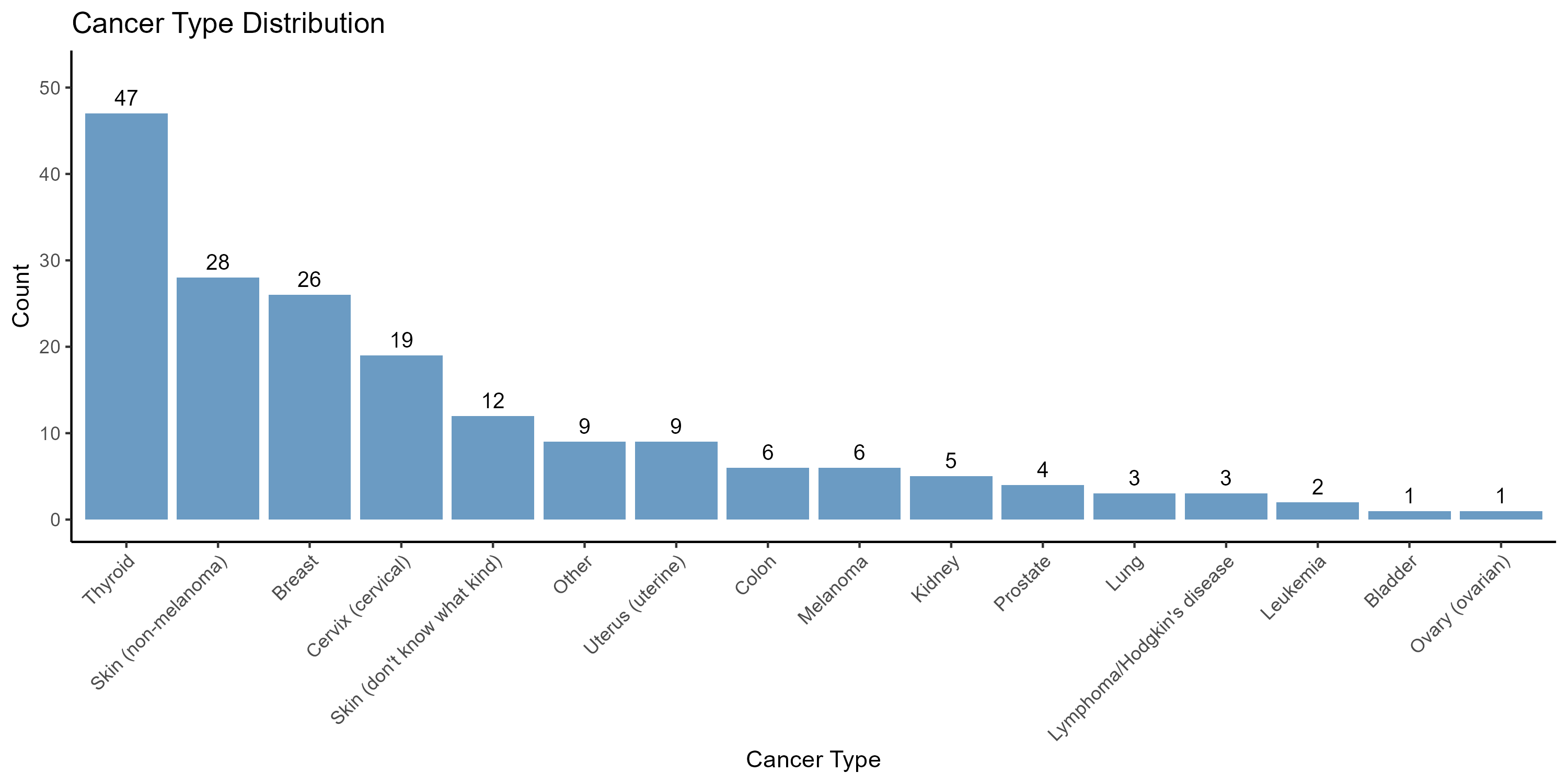

Supplement: S1 Fig — (TIF) [file pone.0341063.s001.tif]

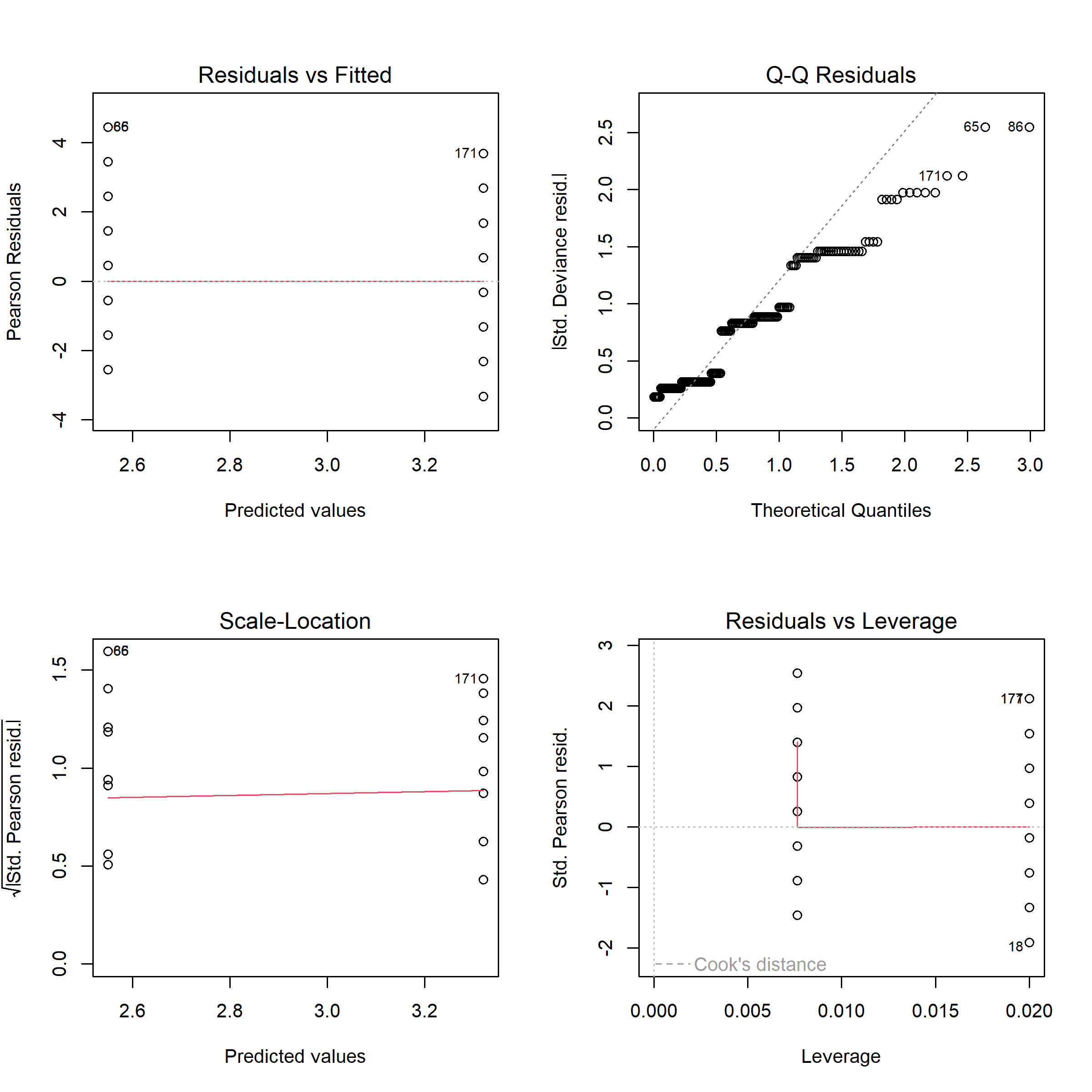

Supplement: S2 Fig — (TIFF) [file pone.0341063.s002.tiff]

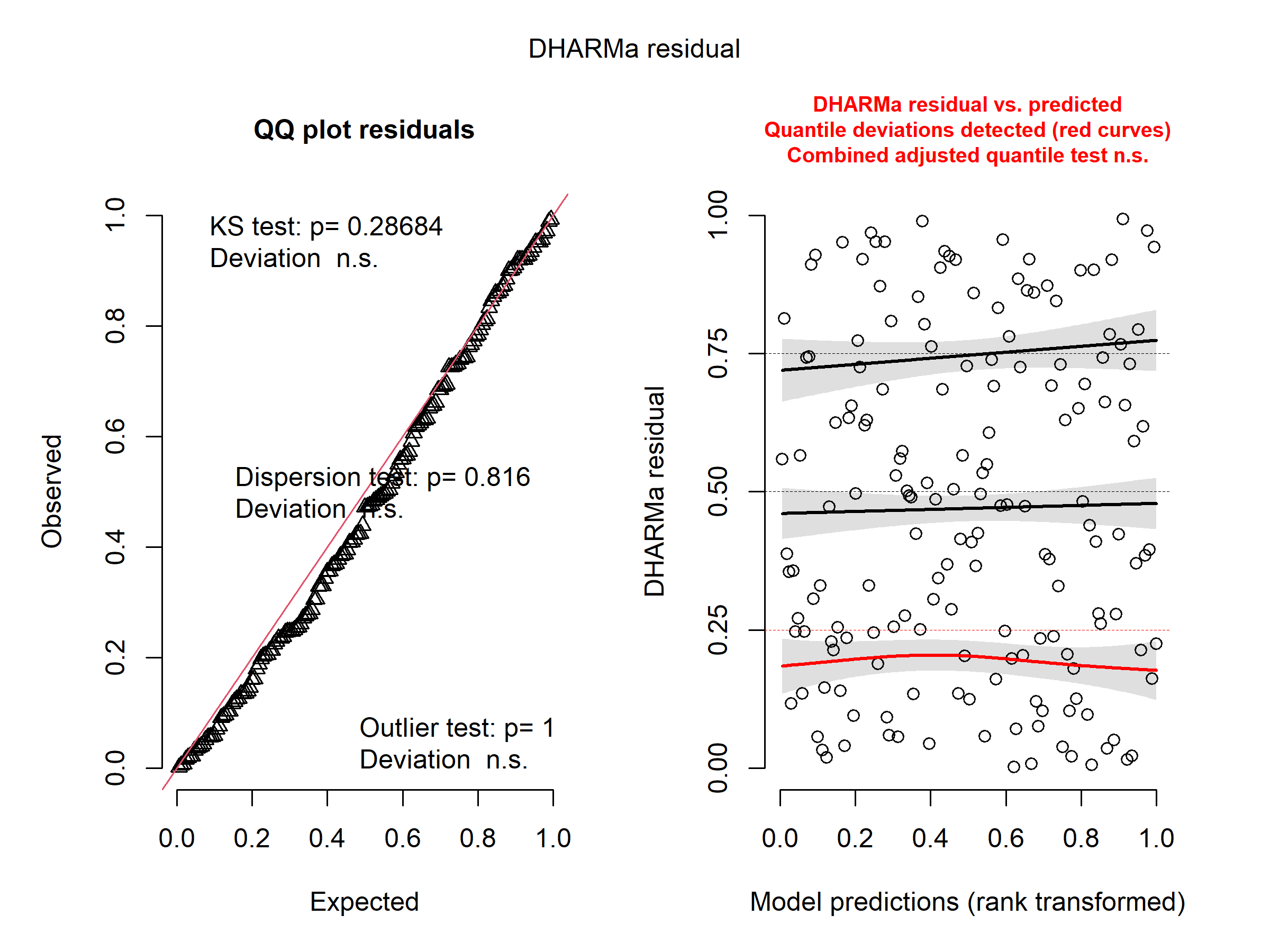

Supplement: S3 Fig — (TIFF) [file pone.0341063.s003.tiff]
