# Supplementary material for: Allostatic load in thyroid cancer is higher than that of other cancers: A secondary analysis using NHANES
Source: PLoS One. 2026 Jan 22;21(1):e0341063. doi: 10.1371/journal.pone.0341063 (PMC12826484; doi:10.1371/journal.pone.0341063)
Supplement: S1 Table — (DOCX) [file pone.0341063.s004.docx]

**S1 Table** Time information for 9 patients with thyroid cancer in this study from the 2007-2008 cycle

| ID | Cancer | Age at MEC | Age at diagnosis |
| --- | --- | --- | --- |
| 41735 | thyroid | 64 | 28 |
| 43358 | thyroid | 71 | 61 |
| 43718 | thyroid | 61 | 55 |
| 44252 | thyroid | 58 | 53 |
| 44668 | thyroid | 46 | 36 |
| 45951 | thyroid | 58 | 57 |
| 46315 | thyroid | 76 | 70 |
| 46914 | thyroid | 44 | 27 |
| 50174 | thyroid | 24 | 20 |
