# Supplementary material for: Allostatic load in thyroid cancer is higher than that of other cancers: A secondary analysis using NHANES
Source: PLoS One. 2026 Jan 22;21(1):e0341063. doi: 10.1371/journal.pone.0341063 (PMC12826484; doi:10.1371/journal.pone.0341063)
Supplement: S2 Table — (DOCX) [file pone.0341063.s005.docx]

**S2 Table** Multicollinearity analysis between variables

| Variables | VIF |
| --- | --- |
| Thyroid cancer | 3.302 |
| age | 1.223 |
| sex | 1.251 |
| race | 1.139 |
| edu | 1.327 |
| marital | 1.270 |
| PIR | 1.477 |
| MCHC | 1.492 |
| RDW | 1.464 |
| diabetes | 1.143 |
| drinking | 1.120 |
| Thyroid disease | 3.315 |
